# WITHDRAWAL: Quantitative modeling of approved and emerging therapeutics for modifying TTR levels in patients with Transthyretin Amyloid Cardiomyopathy

**DOI:** 10.1002/psp4.13078

**Published:** 2024-06-19

## Abstract

Withdrawal: McGirr, K., Sarkar, S., Subramanian, K. (2023) “Quantitative modeling of approved and emerging therapeutics for modifying TTR levels in patients with Transthyretin Amyloid Cardiomyopathy” *CPT: Pharmacometrics & Systems Pharmacology*. https://doi.org/10.1002/psp4.13078.

The above article, published online on November 6, 2023, in Wiley Online Library (http://wileyonlinelibrary.com), has been withdrawn by agreement between the authors, journal Editor‐in‐Chief, France Mentré, the American Society for Clinical Pharmacology and Therapeutics (ASCPT), and Wiley Periodicals LLC. The withdrawal has been agreed to at the authors' request due to a disagreement over the ownership of some data contained therein.

